# Redox post-translational modifications and their interplay in plant abiotic stress tolerance

**DOI:** 10.3389/fpls.2022.1027730

**Published:** 2022-10-26

**Authors:** José M. Martí-Guillén, Miriam Pardo-Hernández, Sara E. Martínez-Lorente, Lorena Almagro, Rosa M. Rivero

**Affiliations:** ^1^ Department of Plant Nutrition, Centro de Edafología y Biología Aplicada del Segura, Consejo Superior de Investigaciones Científicas, Murcia, Spain; ^2^ Department of Plant Biology, Faculty of Biology, University of Murcia, Murcia, Spain

**Keywords:** climate change, abiotic stress, plant tolerance, ROS, RNS, RSS, post-translational modifications, redox regulation

## Abstract

The impact of climate change entails a progressive and inexorable modification of the Earth’s climate and events such as salinity, drought, extreme temperatures, high luminous intensity and ultraviolet radiation tend to be more numerous and prolonged in time. Plants face their exposure to these abiotic stresses or their combination through multiple physiological, metabolic and molecular mechanisms, to achieve the long-awaited acclimatization to these extreme conditions, and to thereby increase their survival rate. In recent decades, the increase in the intensity and duration of these climatological events have intensified research into the mechanisms behind plant tolerance to them, with great advances in this field. Among these mechanisms, the overproduction of molecular reactive species stands out, mainly reactive oxygen, nitrogen and sulfur species. These molecules have a dual activity, as they participate in signaling processes under physiological conditions, but, under stress conditions, their production increases, interacting with each other and modifying and-or damaging the main cellular components: lipids, carbohydrates, nucleic acids and proteins. The latter have amino acids in their sequence that are susceptible to post-translational modifications, both reversible and irreversible, through the different reactive species generated by abiotic stresses (redox-based PTMs). Some research suggests that this process does not occur randomly, but that the modification of critical residues in enzymes modulates their biological activity, being able to enhance or inhibit complete metabolic pathways in the process of acclimatization and tolerance to the exposure to the different abiotic stresses. Given the importance of these PTMs-based regulation mechanisms in the acclimatization processes of plants, the present review gathers the knowledge generated in recent years on this subject, delving into the PTMs of the redox-regulated enzymes of plant metabolism, and those that participate in the main stress-related pathways, such as oxidative metabolism, primary metabolism, cell signaling events, and photosynthetic metabolism. The aim is to unify the existing information thus far obtained to shed light on possible fields of future research in the search for the resilience of plants to climate change.

## 1 Introduction

Climate change is a global and current phenomenon that is changing the Earth’s climate. The main impact of this phenomenon is the increase in the planet global temperature and the modification of rainfall patterns, among other negative effects. The main consequences are manifested in agricultural production, where annual, millionaire economic losses are estimated to progressively increase (Ali et al., 2017; Martinez et al., 2018).

Plants are exposed to abiotic stresses throughout their life cycle, such as salinity, drought, extreme temperatures and heavy metal toxicity, and biotic stresses, induced by organisms such as yeasts, fungi or viruses. We must also add that the different stresses act in nature in a combined way, giving rise to even greater damage. As plants are sessile organisms, their acclimatization and consequent tolerance to simple or combined stress exposure imply physiological, metabolic and molecular restructurings that allow their survival in this type of environment (Ahuja et al., 2010; Rivero et al., 2014; Ahanger et al., 2017; Ma et al., 2020).

These types of modifications allow plants to adapt to the unfavorable environments where they grow, and increase their survival rate. One of the first and fastest plants responses to harsh environments involves an overproduction of reactive chemical species, such as reactive oxygen (ROS), nitrogen (RNS) and sulfur (RSS) species. These molecules have a dual activity; under normal physiological conditions, their production is linked to cell signaling processes, however, under stress conditions, these are massively produced and accumulated (Zhou et al., 2022). The increase in the amounts of these molecules disturbs redox homeostasis, inducing damage to biomolecules and cellular components, such as membranous structures, proteins, nucleic acids, photosynthetic pigments, carbohydrates, hormones, and may even lead to cell destruction due to the oxidative, nitrosative or nitroxidative stress generated, which may trigger cellular apoptosis (Mittler, 2002; Møller et al., 2007; Bose et al., 2014; Martinez et al., 2018; Zhou et al., 2022). In its attempt to survive, the cell tries to buffer this oxidative stress by synthesizing and activating antioxidant enzymes, such as superoxide dismutases, ascorbate peroxidases, catalases, glutathione peroxidases and peroxyredoxins, and antioxidant molecules such as ascorbic acid, glutathione (GSH), melatonin, phenolic compounds, flavonoids, alkaloids and non-protein amino acids. RNS can be detoxified by enzymes such as phytoglobins, peroxyredoxins, tocopherols, flavonoids, ascorbic acid, molecular oxygen, and GSH. In contrast, RSS has a dual behavior with respect to ROS and RNS. These species are overproduced under stress conditions and, although they can modify biomolecules such as protein residues, they actively participate in the detoxification of ROS and RNS, acting as an extra molecular antioxidant system, decreasing the amounts of these two types of reactive species (Wilson et al., 2008; Suzuki et al., 2011; Vaahtera et al., 2014; Gupta and Igamberdiev, 2016; Arnao and Hernández-Ruiz, 2019; Hasanuzzaman et al., 2020; Pardo-Hernández et al., 2020; Paul and Roychoudhury, 2020; Pardo-Hernández et al., 2021; Matamoros and Becana, 2021; Martínez-Lorente et al., 2022).

The balance between the increase in amount reactive species and their detoxification is vital for plant survival. However, cell signaling events are complemented by other types of plant molecular responses. In the process of acclimatization and tolerance to stress conditions, an overproduction of reactive species modulates the expression of stress responses-related genes, and can also lead to the modification of key protein residues to restructure cellular metabolism (Martinez et al., 2018; Nadarajah, 2020; Matamoros and Becana, 2021). ROS, RNS and RSS have the capacity to generate post-translational modifications (PTMs) in the target proteins, some of which are reversible, while others are irreversible. These redox-based PTMs alter the physiological properties of the affected proteins, modifying their structure, function, stability and affinity with other related proteins, biomolecules or metabolites. The vast majority of PMTs are highly regulated and specific to each protein, and are able to enhance its activity, inhibit its function, protect critical residues against catalytic activity or promote its degradation by proteolytic mechanisms (Waszczak et al., 2015; Matamoros and Becana, 2021; Zhou et al., 2022).

Due to the importance of the different ROS, RNS and RSS on cellular metabolism and the functions they play in the acquisition of plant stress tolerance, this review aims to collect the abundant but very dispersed knowledge generated in this scientific field in the last decades, to try to tackle what is currently known, starting from its biogenesis in plant cells, to its main functions and consequences, with special emphasis on the interplay between ROS, RNS, RSS, modification of main cellular components, redox-based PTMs of proteins, and their relationship with the induction of the plants’ tolerance mechanisms to abiotic stress.

## 2 Reactive species: Biogenesis, interplay and antioxidant defense

### 2.1 Biogenesis

Reactive species of a chemical element are molecules that, as a result of their redox state, have a high capacity to react with cellular biomolecules. The most studied types are reactive oxygen (ROS), nitrogen (RNS) and sulfur (RSS) species (Gruhlke and Slusarenko, 2012; Del Río, 2015; Choudhury et al., 2017). Their biosynthesis takes place under any physiological condition (with or without stress), although under stress conditions, their production increases, and therefore their ability to initiate different cell signaling events and redox reactions with cellular components also increases (Brannan, 2010).

**Figure 1:**
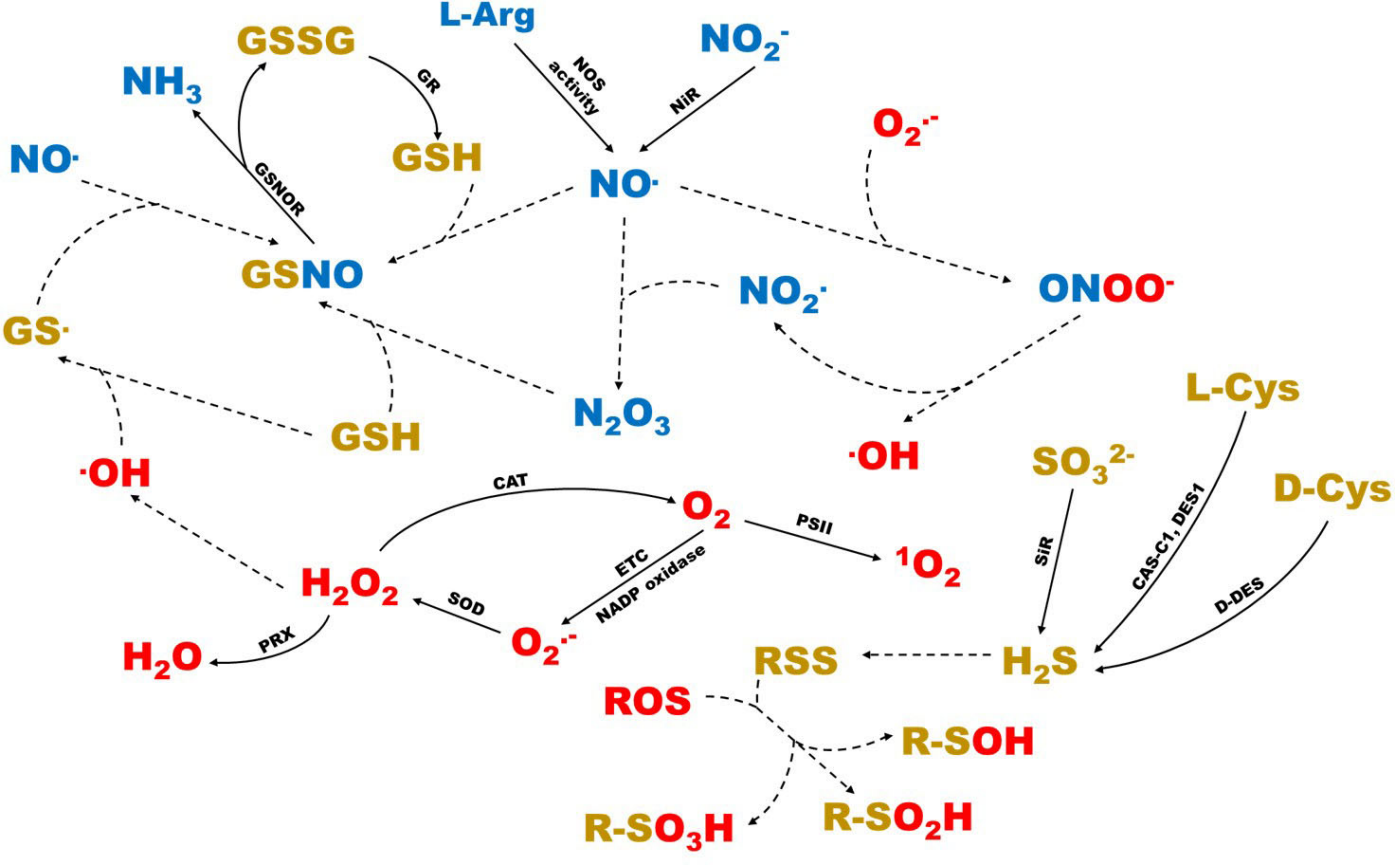
Schematic representation of the interactions between the different ROS, RNS and RSS in the plant cell. In red, the different types of ROS are shown. In blue, the different types of RNS are shown. In gold color, the different types of RSS are shown. Dashed arrows represent reactions that take place non-enzymatically. CAS-C1, cyanoalanine synthase c1; CAT, catalase; D-DES, D-cysteine desulfhydrase; DES1, L-cysteine desulfhydrase 1; ETC, electronic transport chains; GR, glutathione reductase; GSNOR, S-nitrosoglutathione reductase; NiR, nitrite reductase; PRX, peroxidase; PSII, photosystem II; SiR, sulfite reductase; SOD, superoxide dismutase.

ROS are intermediates originating from atmospheric oxygen (O_2_) with a different redox state. There are several types, with different reactivity and oxidizing capacities, among which we find hydrogen peroxide (H_2_O_2_), superoxide anion (O_2_^.-^), singlet oxygen (^1^O_2_), and hydroxyl radical (·OH) (Mittler et al., 2011; Choudhury et al., 2017; Huang et al., 2019a). In plants, they are produced in varying proportions depending on the plant tissue and stage of development, in cellular organelles such as chloroplasts, mitochondria and peroxisomes (**Figure 1**), as well as cellular spaces such as the apoplast, through metabolic pathways where oxidase and peroxidase enzymes participate (Suzuki et al., 2011; Vaahtera et al., 2014; Janků et al., 2019). ^1^O_2_ is produced in photosynthetic metabolism, as a result of the different excitation states of chlorophylls and the activity of reaction centers of photosystem II (Triantaphylidès and Havaux, 2009; Roach and Krieger-Liszkay, 2014; Foyer, 2018; Janků et al., 2019). O_2_^.-^ is generated as a result of electronic transport in mitochondrial and photosynthetic chains, as well as by NADPH membrane oxidases (RBOHs) (Choudhury et al., 2017; Huang et al., 2019a; Matamoros and Becana, 2021). H_2_O_2_ is the most stable ROS, and has an intracellular diffusion capacity (Kim et al., 2018; Arnao and Hernández-Ruiz, 2019). It is generated by the activity of superoxide dismutase (SOD) as a consequence of the extinction of O_2_^.-^ (Foyer, 2018; Janků et al., 2019).OH is the most reactive and unstable ROS, produced as a result of the cleavage of the double bond of H_2_O_2_ by means of the Fenton reaction (Arnao and Hernández-Ruiz, 2019; Huang et al., 2019a). It has the ability to react with many types of biomolecules that are very close to the microenvironment where it is generated (Hiramoto et al., 1996; Kärkönen and Kuchitsu, 2015; Huang et al., 2019a).

RNS are highly reactive nitrogenous derivatives originating from nitric oxide (NO·), as a consequence of oxidative metabolism. The most common and studied species are NO·, nitrogen dioxide (NO_2_.) and nitrogen oxide (III) (N_2_O_3_) (Vandelle and Delledonne, 2011; Arnao and Hernández-Ruiz, 2019). They can generate in the cytosol, as well as in organelles such as mitochondria, chloroplasts and peroxisomes (**Figure 1**), or through enzymatic and non-enzymatic mechanisms (Zhao, 2007; Gupta and Igamberdiev, 2016; Astier et al., 2018; Pardo-Hernández et al., 2020; Matamoros and Becana, 2021). NO·OH can be created from NO_2_^-^ by nitrite reductase (NiR) enzyme, in the apoplast, chloroplasts, mitochondria, and cytoplasm, as well as from L-arginine, by L-arginine-dependent NO synthase (NOS) activity, in mitochondria, peroxisomes, chloroplasts, and cytoplasm (Wilson et al., 2008).

RSS are H_2_S-derived molecules, among which we find the bisulfide anion (HS^-^), radical thiyl (HS.), persulfides (RSSH) and polysulfides (RS(S)_n_H), among others (Gruhlke and Slusarenko, 2012; Corpas et al., 2020; Fukudome et al., 2020). The biosynthesis of H_2_S takes place in all cell organelles and the cytosol, by means of enzymes such as sulfite reductase (SiR), cyanoalanine synthase c1 (CAS-C1), L-cysteine desulfhydrase 1 (DES1), D-cysteine desulfhydrase and Nifs-like proteins (ABA3) (Aroca et al., 2018). Its oxidation results in the formation of thiyl radicals, which are able to establish disulfide bridges with other sulfur atoms, with the consequent formation of the different RSS (Corpas and Barroso, 2015).

### 2.2 Redox-based modifications of main cellular components

#### 2.2.1 Lipids

Lipids, and especially polyunsaturated fatty acids (PUFAs), are susceptible to ROS-mediated oxidative modifications and RNS-mediated nitrosative modifications. ROS-induced modifications, such as ·OH and ^1^O_2_, are generated mainly in galactolipids and phospholipids, resulting in the formation of lipid hydroperoxides that disturb the fluidity of membranes, increasing their permeability and damaging the proteins present within them (Mueller, 2004; Møller et al., 2007). RNS-induced modifications, such as NO· and ONOO^-^, generate nitro-fatty acids, molecules that have been recently described as a result of the interaction between PUFA and RNS. In plants, nitro-oleic acid and nitro-linolenic acid have been mainly characterized, with the latter having the ability to modulate the expression of salinity abiotic stress-related, Cd^2+^ toxicity and wounding genes in *Arabidopsis*. In addition, other properties described for these nitro-fatty acids include their role as NO· donors, with the possibility of inducing RNS-based PTMs on specific proteins (Begara-Morales et al., 2019; Mata-Pérez et al., 2020; Begara-Morales et al., 2021), which will be described in more detail later.

#### 2.2.2 DNA and RNA

ROS and RNS have the ability to modify nucleic acids by attacking the nitrogenous bases that compose them, especially guanine (G) (Møller et al., 2007; Chmielowska-Bąk et al., 2019). ROS, specifically.OH and ^1^O_2_, modify the G bases to 8-hydroxydeoguanosine (8-OH-dG) in DNA and 8-hydroxyguanosine (8-OH-G) in RNA. The presence of these modifications in DNA induces mutations by base pairing error, as well as erroneous copies during the replication process (Møller et al., 2007; Poetsch, 2020; Huang et al., 2021). In addition, the presence of these oxidations can affect the methylation pattern of cytosines, involved in the regulation of gene expression (Halliwell, 2006). In RNA and mRNA, the appearance of oxidized nitrogenous bases is linked to their instability and premature degradation, as well as ribosomal blocking of translation (Simms et al., 2014; Chmielowska-Bąk et al., 2019; Katsuya-Gaviria et al., 2020; Sano et al., 2020). RNS, especially ONOO^-^, modify G bases in DNA and RNA to 8-nitrodeoguanosine (8-NO_2_-dG) and 8-nitroguanosine (8-NO_2_-G), respectively, and GTP nucleotides to 8-NO_2_-GTP molecules (Liu et al., 2012a; Chmielowska-Bąk et al., 2019; Petřivalský and Luhová, 2020). These modifications, specifically in the coding mRNAs, obstruct the correct reading by the ribosome, generating incomplete translations and truncated proteins, with the consequent lack of function, as is the case with oxidation by ROS (Chmielowska-Bąk et al., 2019).

#### 2.2.3 Carbohydrates

It is known that some ROS, especially.OH, have the ability to react with free carbohydrates, such as sugars and polyols, and with structural cell wall polysaccharides. Studies suggest that its reaction with free sugars, such as mannitol, results in an antioxidant protection mechanism, preventing the ·OH reaction with more important cellular biomolecules, thus avoiding superior oxidative damage (Shen et al., 1997; Fry, 1998; Møller et al., 2007). However, indirectly, high oxidative stress conditions induce autoxidation of monosaccharides, with the consequent formation of dicarbonyls, especially glyoxal, methylglyoxal, and 3-deoxyglucosone. These reactive molecules can modify protein residues of Arg and Lys, generating glycation, a PTM linked to enzymatic inactivation (Chaplin et al., 2019; Rabbani et al., 2020; Matamoros and Becana, 2021).

#### 2.2.4 Proteins

The 20 proteinogenic amino acids differ in their chemical reactivity and susceptibility to modifications and hosting any type of PTM. However, most are susceptible to modifications, either spontaneous or enzymatic (Friso and van Wijk, 2015). Redox-based PTMs occur spontaneously, depending on the chemical reactivity of the amino acids susceptible to this type of modification, as well as the concentration of ROS, RNS and RSS, cellular antioxidant capacity, among other factors (Ryšlavá et al., 2013; Friso and van Wijk, 2015). Among these amino acids, those that contain sulfur stand out, especially those with reactive thiol groups: cysteine and methionine. Sulfur presents a wide range of oxidation states that allows different PTMs on the residues that contain it (Rinalducci et al., 2008; Akter et al., 2015; Waszczak et al., 2015). However, not all residues are equally susceptible to modification. This will depend on their dissociation constant (pKa), which in turn will be determined by the microenvironment of the residues: exposure and accessibility, amino acid sequence that flanks them, and local pH, among others (Go et al., 2015; Paul and Roychoudhury, 2020; Matamoros and Becana, 2021). Along with cysteine and methionine, other amino acids are susceptible to modification, such as tyrosine, tryptophan, threonine, lysine, arginine, and proline. However, the susceptibility of these with respect to sulfur-containing amino acids is much lower, so their modification will depend on their interaction with more unstable and aggressive reactive species (Rinalducci et al., 2008; Bizzozero, 2009; Ehrenshaft et al., 2015; Matamoros and Becana, 2021).

### 2.3 Interplay between the different reactive species

In addition to the independent cellular functions described above for ROS, RNS and RSS have the ability to react with each other, generating mixed reactive species. From the interaction between ROS and RNS, reactive species such as peroxynitrite (ONOO^-^) arise, originated by the reaction between NO· and O_2_^.-^, in any cellular space where the generation and coexistence of O_2_^.-^ and NO· occurs, respectively. It is a highly oxidizing molecule, whose decomposition generates NO_2_. as a product (Vandelle and Delledonne, 2011). The interaction between RNS and RSS can produce reactive species such as S-nitrosoglutathione (GSNO), which originates from the reaction between NO· and GSH. It is a mixed reactive species, considered a cellular reservoir of NO·, which is broken down by the NADH-dependent activity of the GSNO reductase (GSNOR) enzyme, into GSSG and NH_3_ (Airaki et al., 2011; Zechmann, 2020). Lastly, the interaction between ROS and RSS can lead to the creation of reactive species such as sulfenic acids (R-SOH), from the reaction between the thiol groups and H_2_O_2_. These molecules are able to react with RSS, generating disulfide bridges, or react with ROS to generate sulfinic acids (R-SO_2_H) and sulfonic acids (R-SO_3_H) (Hasanuzzaman et al., 2020; Matamoros and Becana, 2021). Similarly, some ROS, such as the ·OH radical, can react with GSH, with the consequent formation of the radical thyil, which can react again with NO· for GSNO training (Begara-Morales et al., 2019). All these interactions and the consequent signaling processes take place in the different cellular organelles, with the cytosol being the cellular space where all the signaling triggered by reactive species is integrated (Noctor and Foyer, 2016). These interactions are shown, schematically, in **Figure 1**, which includes all the knowledge available on reactive species.

## 3 ROS, RNS and RSS dependent PTMs

### 3.1 PTMs induced by ROS

#### 3.1.1 Cysteine oxidation

**Figure 2:**
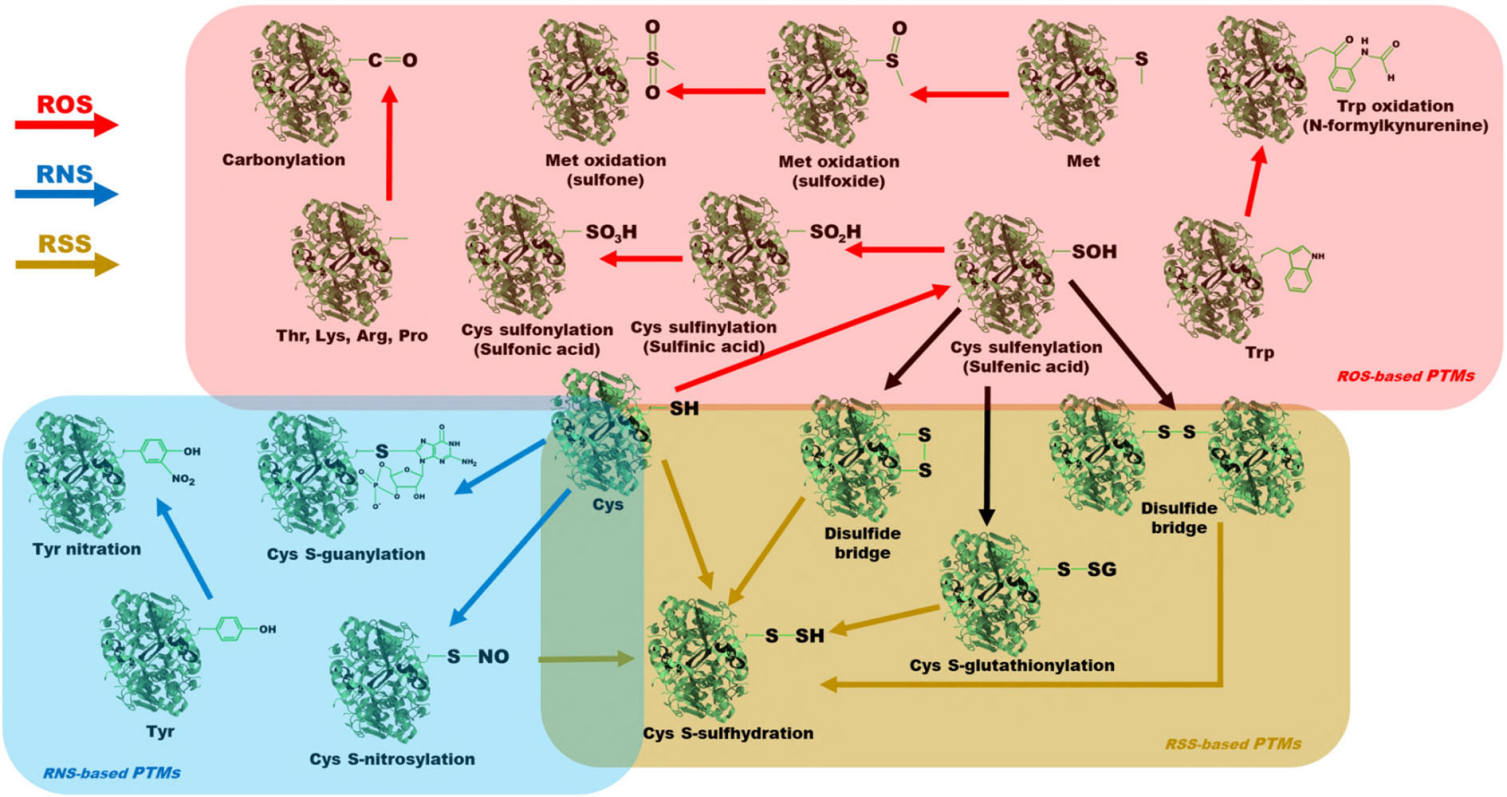
Schematic representation of the different redox-based PTMs of the different protein residues. In red, we find the different ROS-based PTMs: cysteine oxidation, methionine oxidation, tryptophan oxidation and carbonylation. In blue, the different RNS-based PTMs are shown: cysteine S-nitrosylation, cysteine S-guanylation and tyrosine nitration. In gold, the different RSS-based PTMs are indicated: cysteine S-sulfhydration.

The oxidation of cysteine residues (Cys) is mediated by H_2_O_2_, and is linked to the protein structure folding stabilization and its catalytic activity modification (Bigelow and Squier, 2011). H_2_O_2_ reacts with the amino acid thiol group to generate sulfenic acid, in a sulfenylation process (**Figure 2**). Sulfenic acid can react, again, with H_2_O_2_ or with other thiols. The reaction of sulfenic acid with H_2_O_2_ leads to the formation of sulphinic acid, which can, in turn, react again with H_2_O_2_, with the consequent formation of sulfonic acid, in sulfinylation and sulfonylation processes, respectively (Matamoros and Becana, 2021). In contrast, the reaction between sulfenic acid and other thiols can generate intramolecular disulfide bridges and intermolecular disulfide bridges, and the latter may occur with different proteins or with the GSH reactive thiol (S-glutathionylation) (Friso and van Wijk, 2015; Matamoros and Becana, 2021). This type of oxidation and its different modifications affect the functionality of the enzyme that has them, especially if the Cys residues participate in structural stability by means of intramolecular disulfide bridges, or in the enzyme catalytic center (Huang et al., 2019b). The reactions described are schematically depicted in **Figure 2**. All of these PTMs are reversible by enzymes such as glutaredoxins, sulfiredoxins and thioredoxins, except for sulfonic acid formation, which is classified as irreversible and which entails the loss of function of the protein that has it and its consequent degradation (Rey et al., 2007; Meyer et al., 2009; Sevilla et al., 2015; Zhou et al., 2020; Matamoros and Becana, 2021).

#### 3.1.2 Methionine oxidation

Similar to Cys, methionine (Met) residues are susceptible to oxidation especially by H_2_O_2_ (Vogt, 1995). This modification generates Met sulfoxides, resulting in the generation of sulfones if exposure to ROS is relatively high and prolonged (**Figure 2**) (Rinalducci et al., 2008). Met sulfoxides production is a type of PTM that is reversible by a family of Met sulfoxide reductases-mediated mechanisms. However, sulfones are listed as an apparently irreversible PTM (Rouhier et al., 2006; Tarrago et al., 2009; Friso and van Wijk, 2015), although more research is needed on this subject.

The oxidation of Met, which is exposed on three sides in the outer part of the protein and therefore more susceptible to oxidative attack, has a mild effect on the proteins’ structure and function in which it is produced. In fact, some studies suggest that these oxidized residues act as an antioxidant defense mechanism against ROS, protecting catalytic centers and structural domains whose alteration could affect protein function (Levine et al., 1996; Rinalducci et al., 2008). However, a protein with a high percentage of its Met residues oxidized can have its hydrophobicity modified, making it polar and hydrophilic, with its function irreversibly compromised (Rinalducci et al., 2008; Hardin et al., 2009).

#### 3.1.3 Tryptophan oxidation

Tryptophan residues (Trp) are susceptible to ROS modification, especially by ·OH and ^1^O_2_ (Rinalducci et al., 2008; Ehrenshaft et al., 2015). The oxidative attack breaks the ring of its chemical structure and generates N-formylkynurenine, a PTM classified as irreversible, which leads to the proteolysis of the enzyme that presents it (Shacter, 2000; Møller and Kristensen, 2006; Rinalducci et al., 2008). As with Met residues, some research suggests that exposure of Trp residues on the protein surface and its consequent oxidation could act as an antioxidant pathway for the protection of other critical residues needed for the proper functioning of the protein (Levine et al., 1996; Rinalducci et al., 2008).

#### 3.1.4 Carbonylation

Carbonylation is a PTM generated by the reaction between ·OH and certain protein amino acids, such as threonine, lysine, arginine, or proline (**Figure 2**) (Møller et al., 2011; Friso and van Wijk, 2015). It is classified as an irreversible PTM that causes the loss of function of the protein in which it is found (Nyström, 2005; Lounifi et al., 2013). The fate of these proteins is generally degradation by cellular proteolytic complexes (Polge et al., 2009). However, it has recently been described that an excess of carbonylation can generate cytotoxic protein aggregates that, in animals, are linked to aging and some important diseases (Nyström, 2005; Bizzozero, 2009). In plants, carbonylation occurs in response to abiotic stress in proteins found in multiple cellular compartments, including mitochondria, chloroplasts and cytosol, as well as in different metabolic pathways, especially in photosynthetic metabolism and its complementary pathways (Calvin-Benson cycle and photorespiration), whose carbonylated enzymes are inactivated (Tanou et al., 2009; Mano et al., 2014; Smakowska et al., 2014; Friso and van Wijk, 2015; Matamoros and Becana, 2021). Examples of this type of modification are detailed in later sections.

### 3.2 PTMs induced by RNS

#### 3.2.1 Cysteine S-nitrosylation

In the literature, it is common to find the terms “nitrosation” or “nitrosylation” described as a chemical or biochemical reactions involving NO· and, occasionally, both terms are used to describe the same reaction with no distinction between “nitrosation”and/or “nitrosylation”. To this respect, Heinrich et al. (2013) published an excellent review distinguishing between both terms. In this sense, Heinrich et al. (2013) described “nitrosation” as a reaction between a nucleophilic group and an electrophilic nitrosonium ion (NO^+^) whereas “nitrosylation” is described as the direct addition of NO· to a molecule, which, in biological systems, usually refers to the different cellular macromolecules (Heinrich et al., 2013). In this review, the term “nitrosylation”, as proposed by Heinrich et al. (2013), will be used to describe the processes by which the different protein residues undergo the addition of a NO· group to their structures.

S-nitrosylation is a PTM consisting of the covalent bonding of NO· groups with the reactive thiol groups in Cys residues (**Figure 2**), with the consequent formation of S-nitrosothiols (Puyaubert et al., 2014; Feng et al., 2019). The formation of this PTM is directly mediated by NO·, or by donors of NO· groups, such as GSNO and ONOO^-^, and is catalogued as a reversible PTM through thioredoxins or GSH, in a process known as denitrosylation (Wang et al., 2006; Benhar et al., 2009; Zaffagnini et al., 2013; Feng et al., 2019). This PTM affects protein function, being able to enhance or inhibit its activity, depending on the protein and the residue modified (Feng et al., 2019). Due to its reversibility and its potential to modulate enzymatic activity, this type of PTM has garnered a lot of attention in various studies related to possible mechanisms of plant stress tolerance, so it will be discussed in detail later.

#### 3.2.2 Cysteine S-guanylation

8-NO_2_-GTP, an RNS generated by the reaction between ONOO- and GTP, can undergo a cycling process and become 8-NO_2_-cGMP, which reacts with Cys residues, generating an irreversible PTM called S-guanylation (**Figure 2**) (Sawa et al., 2013; Petřivalský and Luhová, 2020). Under abiotic stress conditions due to excess light, it has been described in *Arabidopsis* that 8-NO_2_-cGMP can react with Cys residues, as well as with H_2_S and persulfides, in a global process involving stomatal closure, and thus, this PTM has been related with a putative plant acclimation response (Friso and van Wijk, 2015; Petřivalský and Luhová, 2020).

#### 3.2.3 Tyrosine nitration

Tyrosine residues (Tyr) are susceptible to covalent modification by RNS in the aromatic ring of their chemical structure. This PTM consists of the addition of a NO_2_. group from the reaction between ONOO^-^ or NO_2_. and the Tyr amino acid, with the consequent formation of 3-nitrotyrosine (**Figure 2**) (Rinalducci et al., 2008; Matamoros and Becana, 2021). This PTM is generally linked to a loss of function of the affected protein and its consequent degradation by proteolytic complexes. 3-nitrotyrosine residues increase the hydrophobicity of the protein that contains them and cause steric impediments that can affect the phosphorylation pattern of the residue, modifying the corresponding transduction pathways (Matamoros and Becana, 2021). The formation of this derivative is classified as irreversible, although it has been reported the existence of some mechanisms drived by denitrase activity that, exceptionally, could reverse it, both in animals and plants (Corpas et al., 2008; Kolbert et al., 2017).

### 3.3 PTMs induced by RSS

#### 3.3.1 Cysteine S-sulfhydration

The reactive thiol groups of Cys residues can be modified to a persulfide group by the nucleophilic attack of HS^-^ (**Figure 2**), in a process called S-sulfhydration or persulfidation (Aroca et al., 2015; Aroca et al., 2017). In addition, as indicated above, thiol groups susceptible to this modification may previously be in different oxidation states, including sulfenic acids, disulfide bridges and Cys S-glutathionylated or S-nitrosylated residues (Filipovic and Jovanovic, 2017; Zhou et al., 2020; Matamoros and Becana, 2021). It is a reversible PTM by means of thioredoxins, which act by increasing or decreasing the catalytic activity of the enzyme that has it, although, characteristically, it can also act as a protection against irreversible oxidation states that lead to the loss of function of the protein or its destruction, such as the formation of sulfonic acids (Filipovic et al., 2018). This PTM, in turn, induces structural and functional modifications of proteins, as well as changes in their subcellular location, which may be important for signaling and acclimatization processes to stress (Mustafa et al., 2009; Paul and Snyder, 2012; Aroca et al., 2017).

## 4 Redox-based PTMs of oxidative metabolism-related proteins

The increase in intracellular reactive species as a result of stress exposure induces an increase in their antioxidant enzymes, in order to avoid irreversible damage to cellular components that compromise plant vitality. These enzymes, in addition, are subject to strict redox regulations, carried out by different PTMs in the residues of their structure, with some presenting multiple residues with redox-based PTMs, even multiple PTMs in the same residue, with similar or completely different functionalities.

In *Arabidopsis*, it has been described that the Cys32 residue of the ascorbate peroxidase (APX) enzyme can host two types of redox-based PTMs, S-nitrosylation and S-sulfhydration. Both PTMs increase the enzyme catalytic activity to detoxify H_2_O_2_, affording the plant a greater tolerance to ROS-induced oxidative stress (Aroca et al., 2015; Yang et al., 2015).

In pea, it has been characterized that its homologous enzyme houses several types of redox-based PTMs in its amino acid sequence, with different consequences on its catalytic activity. The Cys32 residue, included in the APX binding site, can be S-nitrosylated, with the consequent increase in the scavenger catalytic activity of H_2_O_2_. On the other hand, the Tyr235 residue, included in the pocket that encloses the heme group, can be irreversibly nitrated by ONOO^-^, with the consequent enzymatic inactivation (Begara-Morales et al., 2014).

In addition, in *Arabidopsis*, the dehydroascorbate reductase 2 (DHAR2) enzyme can undergo two PTMs on its Cys20: Cys oxidation and Cys S-glutathionylation. H_2_O_2_ can cause an overoxidation of this Cys towards sulfenic and sulfonic acids, with the consequent decrease in enzyme activity. However, Cys20 may be S-glutathionylated, a mechanism by which covalent GSH binding protects the residue from irreversible overoxidation, suggesting a crucial role of Cys20 in the DHAR2 catalytic activity (Waszczak et al., 2014).

In pea, the monodehydro-ascorbate reductase (MDHAR) enzyme can be nitrated by ONOO^-^ in its Tyr345 residue, three-dimensionally close to the His313 residue, included in the NADP binding site. This Tyr345 nitration is associated with a decrease in the enzyme catalytic activity (Begara-Morales et al., 2015; Kolbert et al., 2017).

In the *Antiaris toxicaria* tree, the ascorbate-glutathione relatedenzymes, involved in H_2_O_2_ detoxification, are also subject to redox regulation. These enzymes, such asAPX, MDHAR, DHAR and GR, undergo a carbonylation reaction with their consequent inactivation when the cellular H_2_O_2_ concentrations are high, as occurs under desiccation conditions (Bai et al., 2011). However, when plants were treated with NO·, these enzymes undergo S-nitrosylation with the concomitant increase in their catalytic activity. Therefore, both mechanisms can be described as antagonistics by which the cellular balance between H_2_O_2_ and NO· will control the activation/deactivaton of these enzymes (Bai et al., 2011).

In *Arabidopsis*, the GSNO reductase (GSNOR) enzyme, involved in thermotolerance, redox homeostasis, and NO· metabolism, can be S-nitrosylated in three different Cys residues preserved in plants: Cys10, Cys271 and Cys370. This redox-based PTM reduces the enzyme catalytic activity, with Cys370 being the preferred residue for S-nitrosylation (Guerra et al., 2016). S-nitrosylation has also been described in cucumber´s GSNOR with similar consequences (Niu et al., 2019).

The catalase (CAT) enzyme is subject to redox regulation by means of a multitude of different PTMs, even in different types of plant species. In both pea and *Arabidopsis*, Cys S-nitrosylation decreases the enzyme’s catalytic activity, with this enzyme also inactivated by Cys S-sulfhydration in the latter plant (Ortega-Galisteo et al., 2012; Corpas et al., 2019; Palma et al., 2020). This PTM has also been described for the sweet pepper´s CAT, which is also susceptible to Tyr nitration, with the consequent decrease in its catalytic activity (Chaki et al., 2015; Corpas et al., 2019).

In contrast to other plant species, in tobacco, it has been shown that their exposure to NO· donors reduced the catalytic activity of APX and CAT enzymes Clark et al. (2000) suggested that this could be due to the formation of an iron-nitrosyl intermediate between NO· and the iron atom of the haem prosthetic group of these enzymes, compromising H_2_O_2_ scavenging (Clark et al., 2000).

Among these enzymes, those that use GSH as a substrate are also subject to redox regulation. In *Arabidopsis*, two glutathione-S-transferase enzymes, GSTF9 and GSTT23, are regulated by Met oxidation, with Met35 and Met47, respectively, being the residues where these PTMs have been described, in both cases reducing their catalytic activity (Jacques et al., 2015). In cucumber, it has been described that glutathione reductase (GR) may decrease its activity as a result of Cys S-nitrosylation (Niu et al., 2019).

Superoxide dismutase family (SODs) enzymes are also susceptible to this type of redox regulations. The *Arabidopsis* mitochondrial Mn-SOD (MSD1) enzyme can be nitrated in its Tyr63 residue, located near the active center of the enzyme, which may impede the accessibility of the substrate and, as a consequence, decrease its activity (Holzmeister et al., 2015; Kolbert et al., 2017). Similarly, the Mn-SOD (MSD2) enzyme from *Arabidopsis* can be nitrated in its Tyr68 residue, with similar consequences (Chen et al., 2022).

Yun et al. (2011) described in Arabodopsis a mechanism by which S-nitrosylation at its Cys890 abolished its capacity for ROS synthesis and, therefore, the initiation of the cell death process, governed by ROS, was compromised. This Cys890 and its S-nitrosylation was found to be conserved in the NADPH oxidase RBOHD human and fly homologous enzymes, suggesting that this process could be conserved for the regulation of the cell death (Yun et al., 2011). On the other hand, Romero-Puertas et al. (2007) described that the PrxII E enzyme had peroxynitrite reductase activity, which it was inhibited, along with its peroxidase activity, by NO·-mediated S-nitrosylation of its Cys121. The consequences of this S-nitrosylation were the increase in the cellular concentration of ONOO^-^ with the concomitant increase in proteins that suffered nitration in their Tyr residues, suggesting a signaling mechanism mediated by ONOO^-^ (Romero-Puertas et al., 2007).

In summary, the regulation of metabolism by means of a redox-based PTM can enhance the catalytic activity of an enzyme or decrease it. In turn, it is common to find specific enzymes that contain a multitude of residues susceptible to different redox regulations, as is the case of GSNOR, CAT or APX. This last enzyme, in addition, has a critical residue for its catalytic activity, Cys32, characterized in several plant species, which can present different types of redox-based PTMs, and which have been cataloged as having a special importance in the induction of tolerance to environmental stresses.

The information described on oxidative metabolism-related proteins is shown in **Table 1**.

**Table 1:** PTMs and their effect on the different oxidative metabolism-related proteins subjected to redox regulation.

Plant	Protein (Uniprot accession number)	PTM	Effect	Reference
*Arabidopsis*	**APX** (Q05431)	Cys-32 S-sulfhydration	Increased activity	(Aroca et al., 2015)
* *	**APX** (Q05431)	Cys-32 S-nitrosylation	Increased activity	(Yang et al., 2015)
*Pea*	**APX** (P48534)	Tyr-235 nitration	Decreased activity	(Begara-Morales et al., 2014)
* *	**APX** (P48534)	Cys-32 S-nitrosylation	Increased activity	(Begara-Morales et al., 2014)
*Antiaris*	**APX** (-)	Cys-S-nitrosylation	Increased activity	(Bai et al., 2011)
* *	**APX** (-)	Carbonylation	Decreased activity/Inactivation	(Bai et al., 2011)
*Tobacco*	**APX** (Q42941)	Cys-S-nitrosylation	Decreased activity/Inactivation	(Clark et al., 2000)
*Arabidopsis*	**DHAR2** (Q9FRL8)	Cys-20 oxidation	Decreased activity	(Waszczak et al., 2014)
* *	**DHAR2** (Q9FRL8)	Cys-20 S-glutathionylation	Cys overoxidation protection	(Waszczak et al., 2014)
*Antiaris*	**DHAR** (-)	Cys-S-nitrosylation	Increased activity	(Bai et al., 2011)
* *	**DHAR** (-)	Carbonylation	Decreased activity/Inactivation	(Bai et al., 2011)
*Pea*	**MDHAR** (Q40977)	Tyr-345 nitration	Decreased activity	(Begara-Morales et al., 2015; Kolbert et al., 2017)
*Antiaris*	**MDHAR** (-)	Cys-S-nitrosylation	Increased activity	(Bai et al., 2011)
* *	**MDHAR** (-)	Carbonylation	Decreased activity/Inactivation	(Bai et al., 2011)
*Arabidopsis*	**GSNOR** (Q96533)	Cys-10, Cys-271, Cys-370 S-nitrosylation	Decreased activity	(Guerra et al., 2016)
*Cucumber*	**GSNOR** (A0A0A0KBZ1)	Cys-S-nitrosylation	Decreased activity	(Niu et al., 2019)
*Pea*	**CAT** (P25890)	Cys-S-nitrosylation	Decreased activity	(Ortega-Galisteo et al., 2012)
*Arabidopsis*	**CAT** (Q96528/P25819/Q42547)	Cys-S-sulfhydration	Inactivation	(Corpas et al., 2019)
* *	**CAT** (Q96528/P25819/Q42547)	Cys-S-nitrosylation	Decreased activity	(Palma et al., 2020)
*Sweet pepper*	**CAT** (Q9M5L6)	Cys-S-sulfhydration	Inactivation	(Corpas et al., 2019)
* *	**CAT** (Q9M5L6)	Tyr-nitration	Decreased activity	(Chaki et al., 2015)
*Tobacco*	**CAT** (P49319)	Cys-S-nitrosylation	Decreased activity/Inactivation	(Clark et al., 2000)
*Arabidopsis*	**GSTF9** (O80852)	Met-35 oxidation	Decreased activity	(Jacques et al., 2015)
*Arabidopsis*	**GSTT23** (Q9M9F1)	Met-47 oxidation	Decreased activity	(Jacques et al., 2015)
*Antiaris*	**GR** (-)	Cys-S-nitrosylation	Increased activity	(Bai et al., 2011)
* *	**GR** (-)	Carbonylation	Decreased activity/Inactivation	(Bai et al., 2011)
*Cucumber*	**GR** (A0A0A0K8Q7)	Cys-S-nitrosylation	Decreased activity	(Niu et al., 2019)
*Arabidopsis*	**MSD1** (O81235)	Tyr-63 nitration	Decreased activity	(Holzmeister et al., 2015)
*Arabidopsis*	**MSD2** (Q9LYK8)	Tyr-68 nitration	Inactivation	(Chen et al., 2022)
*Arabidopsis*	**RBOHD** (Q9FIJ0)	Cys-890 S-nitrosylation	Abolishes ROS production	(Yun et al., 2011)
*Arabidopsis*	**PrxII E** (Q949U7)	Cys-121 S-nitrosylation	Decreased peroxidase and peroxynitrite reductase activities	(Romero-Puertas et al., 2007)

## 5 Redox-based PTMs on primary metabolism-related proteins

Primary metabolism plant enzymes, including energy production and amino acid biosynthetic pathways, have also been described as subject to redox regulation.

In *Arabidopsis*, the nitrate reductase (NR) enzyme, involved in nitrate (NO_3_^-^) assimilation, has Met538 a residue, included in a phosphorylation motif, whose Ser534 is subject to phosphorylation/dephosphorylation events. H_2_O_2_-induced oxidation generates Met sulfoxide, a PTM that prevents the Ser538 residue from being phosphorylated (Hardin et al., 2009; Matamoros and Becana, 2021). The cellular persistence of NR depends on the phosphorylation state of Ser534 which, if phosphorylated, generates a canonical motif of a 14-3-3-binding site, allowing the binding of 14-3-3 proteins and their consequent degradation (MacKintosh and Meek, 2001). The binding of 14-3-3 proteins induces a conformational change in NR that prevents electron transfer from the enzyme’s heme group to the molybdenum cofactor (Lambeck et al., 2012). Directed mutagenesis studies of the rice NR enzyme demonstrated how the absence of phosphorylation in the motif increased NO_3_^-^ assimilation efficiencies (Han et al., 2022). This evidence suggests that ensuring the change of NO_3_^-^ to NH_4_^+^ is necessary under stress conditions. However, it does not seem that the generation of NH_4_^+^ is diverted towards the production of amino acids such as glutamine, since it has been observed that the glutamine synthetase (GS) enzyme of *Medicago* is inhibited by Tyr nitration in its Tyr167 residue, just as the homologous GS from *Arabidopsis* decreases its catalytic activity by Cys S-sulfhydration (Melo et al., 2011; Aroca et al., 2015). This persistence in NH_4_^+^ production could be necessary for the biosynthesis of other nitrogenous compounds, such as polyamines, molecules that have been described as participating in the processes of plant abiotic stress tolerance (Pathak et al., 2014; Chen et al., 2019; Marino and Moran, 2019).

The O-acetylserine(thiol)lyase A1 (OASA1) enzyme participates in the sulfur assimilation and biosynthesis of Cys metabolic pathways, and in *Arabidopsis*, the nitration of the Tyr302 residue causes its inactivation, as this residue is close to another key residue, Asn77, found in the O-acetylserine binding site (Álvarez et al., 2011; Vandelle and Delledonne, 2011). Álvarez et al. (2011) propose that the inactivation of OASA1 could represent a rapid regulatory mechanism by which the production of Cys and GSH is limited, localized, and under stress conditions, decreasing its antioxidant capacity and its ability to detoxify other reactive species, thus allowing the transduction of stress signals to the rest of the plant (Álvarez et al., 2011).

Tyr nitration has also been described in the S-adenosyl homocysteine hydrolase (SAHH) from sunflower, with its Tyr448 residue as preferably modifiable, based on *in silico* studies, and whose modification decreases its catalytic activity. This enzyme participates in the genomic DNA methylation cycle, so its inactivation could be a mechanism by which an epigenetic regulation of DNA takes place, with the consequent modification of gene expression in the stress tolerance process (Chaki et al., 2009; Akhter et al., 2021).

In pea, the NADP-isocitrate dehydrogenase (NADP-ICDH) enzyme decreases its activity by Tyr392 nitration, as does the malate dehydrogenase (MDH) enzyme, although in this case, it is through Cys S-nitrosylation (Ortega-Galisteo et al., 2012; Begara-Morales et al., 2013). Both enzymes are involved in energy metabolism and amino acid biosynthesis. The inactivation of NADP-ICDH is attributed to the process of root senescence (Begara-Morales et al., 2013); while in the case of MDH, recent studies have shown that rice plants deficient in plastidial MDH1 activity acquire saline stress tolerance by enhancing the biosynthetic pathway of vitamin B6 (Nan et al., 2020).

The consequences from the different types of redox regulations to which the described enzymes are subject, generally lead to a loss or decrease in their metabolic activity, or may even result in the partial deactivation of complete metabolic pathways, through the total inactivation of key enzymes. This is of vital importance for both the regulation of a specific metabolic pathway and for choosing these molecular targets in the selection and genetic editing of plants resilient to abiotic stress.

The information described on primary metabolism-related proteins can be found in **Table 2**.

**Table 2:** PTMs and their effect on the different primary metabolism-related proteins subject to redox regulation.

Plant	Protein (Uniprot accession number)	PTM	Effect	Reference
*Arabidopsis*	**NR** (P11035)	Met-538 oxidation	Inhibition of phosphorylation at Ser-534	(Hardin et al., 2009; Matamoros and Becana, 2021)
*Medicago*	**GS** (O04999)	Tyr-167 nitration	Inactivation	(Melo et al., 2011)
*Arabidopsis*	**GS** (Q43127)	Cys-S-sulfhydration	Decreased activity	(Aroca et al., 2015)
*Arabidopsis*	**OASA1** (P47998)	Tyr-302 nitration	Inactivation	(Álvarez et al., 2011)
*Sunflower*	**SAHH** (A0A251SAA5)	Tyr-448 nitration	Decreased activity	(Chaki et al., 2009)
*Pea*	**NADP-ICDH** (Q6R6M7)	Tyr-392 nitration	Decreased activity	(Begara-Morales et al., 2013)
*Pea*	**MDH** (Q5JC56)	Cys-S-nitrosylation	Decreased activity	(Ortega-Galisteo et al., 2012)

## 6 Redox-based PTMs on cellular signaling-related proteins

Many important enzymes in cell signaling processes are also susceptible to redox regulation in different plant species. This regulation implies the fine tuning of certain important enzymes, which makes them prone to be included within the target molecules involved in the plants’ tolerance to abiotic stresses. Although the information available in the literature on this subject is limited, it is important to mention some of the most important aspects described by some authors.

In *Arabidopsis*, it has been described that the protein arginine methyltransferase 5 (PRMT5) enzyme, involved in the transfer of methyl groups in arginine residues, can undergo S-nitrosylation in its Cys125 residue during stress responses. Hu et al. (2017) have described a stress tolerance mechanism linked to this PTM. The S-nitrosylation of Cys125 enhances its catalytic activity, inducing an increase in spliceosome methylation levels, causing the alternative processing of pre-mRNAs of genes related to stress tolerance (Hu et al., 2017).

In *Arabidopsis*, the small heat shock protein 21 (sHSP21) chaperone is regulated by oxidation of its Met residues. Met49, Met52, Met55 and Met59 could be oxidized, decreasing their catalytic activity progressively depending on the degree of oxidation, thereby achieving enzymatic inactivation in case of a certain degree of overoxidation (Gustavsson et al., 2002; Jacques et al., 2013).

In beans, the nitration of Tyr30, a residue located in the distal heme pocket of a leghemoglobin (Lb), an enzyme involved in O_2_ transport and present in abundance in legume nodules, inactivates its catalytic activity. The PTM of this enzyme suggests a mechanism by which Lb acts as an ONOO^-^ scavenger for the protection of the symbiotic complex in legumes (Sainz et al., 2015).

As described, it is important to deepen our knowledge on specific PTMs that are induced in important proteins that are part of cell signaling processes, to find targets susceptible to being genetically edited in the generation of plants that are more resilient to climate change.

### 6.1 Phosphorylation/dephosphorylation events

Cell signaling can take place by means of protein phosphorylation/dephosphorylation events, carried out by protein kinases and protein phosphatases. These types of enzymes are susceptible to PTMs, altering their ability to induce the corresponding signaling events, as well as certain phosphorylation/dephosphorylation motifs whose modification may be compromised by another type of redox-based PTM, such as that described for the Met538 residue of the NR enzyme.

In *Arabidopsis*, leucine-rich repeat receptor protein kinase (HPCA1) is a transmembrane receptor that undergoes redox regulation. Oxidation of the Cys421/Cys424 and Cys434/Cys436 residue pairs of HPCA1 by H_2_O_2_, in the extracellular domain of the protein, activates the intracellular kinase domain and induces its autophosphorylation, mediating the activation of Ca^2+^ channels in the process of stomatal closure in guard cells (Wu et al., 2020).

Another characterized example is the protein tyrosine phosphatase 1 (PTP1) enzyme. It is the only Tyr-specific PTP described in *Arabidopsis*, whose activity consists of repressing the production of H_2_O_2_ and regulating the activity of the Mitogen-Activated Protein Kinases (MAPKs) MPK3/MPK6. PTP1 presents a Cys catalytic residue, Cys265, whose irreversible overoxidation induced by H_2_O_2_ inhibits its activity. However, Cys265 may suffer S-nitrosylation, induced by NO·, in a mechanism by which it protects the catalytic residue from overoxidation and consequent enzymatic inactivation (Nicolas-Francès et al., 2022).

Another enzyme, *Arabidopsis* protein kinase SnRK2.6, may be S-nitrosylated in its Cys137 by NO· This residue is found next to the kinase catalytic site, and by housing the PTM, it inhibits its function. This is associated with disturbances in the proper functioning of the ABA-induced stomatal closure (Wang et al., 2015).

S-nitrosylation also takes place in the phosphoinositide-dependent kinase 1 (PDK1) enzyme of tomato. Its Cys128 residue can have this PTM, and the consequences of this modification are the inhibition of its kinase activity, and therefore of the phosphorylation events (Liu et al., 2017).

In maize, the cyclin-dependent kinase A (CDKA;1) enzyme can undergo Tyr nitration in its Tyr15 and Tyr19 residues. Both Tyr are on the ATP-binding site, and their nitration causes a decrease in affinity with ATP (Méndez et al., 2020). Similar events occur in its brassinosteroid insensitive 2 kinase (BIN2) enzyme by NO·-mediated modifications. It is a protein kinase that participates in the brassinosteroid-mediated response, and the S-nitrosylation of Cys162 compromises its assembly, and consequently, its kinase activity (Mao and Li, 2020). However, the BIN2 homologous enzyme from *Arabidopsis* is inactive until, as a consequence of an increase in ROS, its residues Cys59, Cys95, Cys99 and Cys162 undergo oxidation, with the subsequent formation of intramolecular disulfide bridges (Song et al., 2019).

Thus, the above describes some examples of plant enzymes linked to phosphorylation/dephosphorylation cell signaling events. Most redox-based PTMs on protein kinases and phosphatases inhibit their ability to introduce or remove phosphate groups into and from target proteins, modifying the corresponding cell signaling events.

### 6.2 Phytohormone-related signaling events

A part of the overall coordination of plant metabolism corresponds to cell signaling events carried out by phytohormones. These occur under physiological conditions for proper plant development, apical growth, root development and senescence, signaling under biotic stress conditions and pathogenesis, and abiotic stress, including salinity, drought, and extreme temperatures. In recent years, different experimental approaches have described how multiple proteins from the biosynthetic metabolism of these phytohormones and their signaling, are subject to a fine redox regulation.

Abscisic acid (ABA) metabolism and the signaling events in which this phytohormone is involved, require biosynthetic enzymes, receptors from the PYR/PYL/RCAR family, transcription factors, PP2C phosphatases and SnRK2 kinases (Skubacz et al., 2016). Multiple ABA receptors from the PYR/PYL/RCAR family have been described as being under redox regulation by Tyr nitration. Under abiotic stress conditions, the increase in the ABA concentration is associated with the increase in ROS and RNS, with the consequent ONOO^-^ formation. The ABA receptor Pyrabactin resistance 1 (PYR1) shows three Tyr residues with increased susceptibility to ONOO^–^induced nitration. These residues, Tyr58, Tyr120 and Tyr143, are oriented and near the ABA-binding site, interfering in its appropriate binding and representing a mechanism by which nitrosative stress conditions locally limit the effects of ABA signaling (Castillo et al., 2015). In *Arabidopsis*, the transcription factors AtMYB2 and AtMYB30 are induced by abiotic stress. AtMYB2 participates in ABA-induced signaling, and AtMYB30 in the heat and oxidative stress hypersensitive response (Mabuchi et al., 2018; Jia et al., 2020; Pande et al., 2022). Both transcription factors are subject to redox regulation by S-nitrosylation, with Cys53 in AtMYB2, and Cys49 and Cys53 in AtMYB30 being the residues susceptible to modification, consequently decreasing their DNA binding activity (Serpa et al., 2007; Tavares et al., 2014; Pande et al., 2022). Another antagonistic behavior of NO· on ABA involves the transcription factor ABI5, which represses seed germination and growth. ABI5 is susceptible to S-nitrosylation in its residue Cys153, facilitating its degradation through E3 ligases and proteolytic complexes, consequently promoting germination (Albertos et al., 2015). Examples of SnRK2 kinases have been described in previous sections, with the example of SnRK2.6, involved in ABA-induced stomatal closure. These proteolytic complexes, especially the SCF-E3 ligase complex, have also been described as susceptible to S-nitrosylation in the subunits that compose it, enhancing the assembly and stability of the complex with itself and interactions with other proteins (Pande et al., 2022). The ASK1 subunit undergoes S-nitrosylation in its Cys37 and Cys118 residues, a modification that enhances the assembly with the rest of the subunits (Iglesias et al., 2018). The S-nitrosylation of the TIR1 subunit, in its Cys140 residue, enhances the assembly of the complex and is critical for its interaction and ubiquitination with the AUX/IAA repressor, involved in the induction of NO·-modulated auxin responses (Terrile et al., 2012).

The NO· balance has also been described as modulating the cytokinin response, negatively regulating its signaling. Cytokinin promotes increased kinase and phosphotransferase activity of proteins such as histidine phosphotransfer protein 1 (AHP1), an activity that is inhibited by NO·, through the S-nitrosylation of its Cys115 residue, attenuating the induction of the phytohormone response. Therefore, redox balance and the amount of NO· coordinate cytokinin-induced responses, mainly plant growth and development (Feng et al., 2013; Pande et al., 2022).

Different redox-based PTMs regulate the Yang cycle and ethylene biosynthesis. NO· regulates this cycle through modifications in enzymes such as S-adenosylmethionine synthase (SAMS). In *Arabidopsis*, the SAMS enzyme is inactivated by S-nitrosylation of its Cys114 residue (Lindermayr et al., 2006). The 1-aminocyclopropane-1-carboxylate oxidase 1 (ACO1) enzyme of tomato, involved in the initial steps of the ethylene biosynthesis pathway from SAM, undergoes S-sulfhydration in its Cys60 residue, decreasing the enzyme catalytic activity. Tomato’s ACO2 enzyme is also inhibited by S-sulfhydration (Jia et al., 2018). Other types of proteins described under redox regulation are the master regulator transcription factors. In tomato, the master regulator transcription factor NOR, involved in fruit ripening, and acting upstream of ethylene biosynthesis, undergoes oxidation in the Met138 residue, decreasing its affinity for DNA. The decrease in affinity between a transcription factor and its DNA target sequence modifies the gene expression patterns that are under its regulation, and in the case of NOR, ripening-related genes (Jiang et al., 2020). These enzymatic inhibitions or decreased DNA binding activity in the case of transcription factors, decrease ethylene production. It is speculated that other enzymes from the Yang cycle and ethylene biosynthesis are subject to redox regulation, but more studies are needed to elucidate it.

Cell signaling events induced by the main hormones involved in biotic stress by pathogenesis processes, such as salicylic acid (SA) and jasmonic acid (JA), have been described as able to be modulated by NO· in nitrosative stress scenarios. NO· mainly has a negative and attenuating effect of the signaling induced by both phytohormones. This is shown with examples such as the S-nitrosylation of the Cys229 residue of the JAZ1 repressor from *Arabidopsis*, a modification that improves the union between the repressor and its co-repressors, inhibiting JA signaling (Pande et al., 2022). Likewise, in *Arabidopsis*, the increase in NO· induces S-nitrosylation of salicylic acid-binding protein 3 (AtSABP3) in its Cys280 residue, inhibiting its binding to SA and its carbonic anhydrase activity (Wang et al., 2009). NPR1, a pathogenesis master regulator, interacts with the basic leucine zipper TGA transcription factor to induce gene expression linked to the pathogen infection response. Both proteins have been described in *Arabidopsis*, as susceptible to redox-based PTMs. The activity of NPR1 occurs when the protein is in a monomeric state, but its residue Cys156 is susceptible to S-nitrosylation, a modification that enhances the assembly of an oligomeric state in the cytosol, and prevents the development of its function (Tada et al., 2008). Complementarily, other studies have shown that TGA1 is susceptible to S-nitrosylation and S-glutathionylation in its Cys260 and Cys266 residues, both redox-based PTMs protect TGA1 from an overoxidation state, and enhances its DNA binding activity in the presence of NPR1 (Lindermayr et al., 2010).

The information described on cellular signaling-related proteins is summarized in **Table 3**.

**Table 3:** PTMs and their effect on the different cellular signaling-related proteins subject to redox regulation.

Plant	Protein (Uniprot accession number)	PTM	Effect	Reference
*Arabidopsis*	**PRMT5** (Q8GWT4)	Cys-125 S-nitrosylation	Increased activity	(Hu et al., 2017)
*Arabidopsis*	**sHSP21** (P31170)	Met-49, Met-52, Met-55, Met-59 oxidation	Decreased activity/Inactivation	(Gustavsson et al., 2002; Jacques et al., 2013)
*Bean*	**LB** (P02234)	Tyr-30 nitration	Inactivation	(Sainz et al., 2015)
*Arabidopsis*	**HPCA1** (Q8GZ99)	Cys-421/Cys-424 and Cys-434/Cys-436 oxidation	Activation of intracellular kinase activity	(Wu et al., 2020)
*Arabidopsis*	**PTP1** (O82656)	Cys-265 S-nitrosylation	Cys overoxidation protection	(Nicolas-Francès et al., 2022)
* *	**PTP1** (O82656)	Cys-265 oxidation	Inactivation	(Nicolas-Francès et al., 2022)
*Arabidopsis*	**SnRK2.6** (Q940H6)	Cys-137 S-nitrosylation	Inactivation	(Wang et al., 2015)
*Tomato*	**PDK1** (Q5I6E8)	Cys-128 S-nitrosylation	Inactivation	(Liu et al., 2017)
*Maize*	**CDKA;1** (P23111)	Tyr-15, Tyr-19 nitration	Lower affinity to ATP binding	(Méndez et al., 2020)
*Maize*	**BIN2** (-)	Cys-162 S-nitrosylation	Decreased activity	(Mao and Li, 2020)
*Arabidopsis*	**BIN2** (Q39011)	Cys-59, Cys95, Cys-99, Cys-162 oxidation	Activation/Increased activity	(Song et al., 2019)
*Arabidopsis*	**PYR1** (O49686)	Tyr-58, Tyr-120, Tyr-143 nitration	Inactivation	(Castillo et al., 2015)
*Arabidopsis*	**MYB2 TF** (Q9SPN3)	Cys-53 S-nitrosylation	Decreased the DNA binding activity	(Jia et al., 2020)
*Arabidopsis*	**MYB30 TF** (Q9SCU7)	Cys-49, Cys-53 S-nitrosylation	Decreased the DNA binding activity	(Mabuchi et al., 2018)
*Arabidopsis*	**ABI5 TF** (Q9SJN0)	Cys-153 S-nitrosylation	Triggers ABI5 degradation	(Albertos et al., 2015)
*Arabidopsis*	**ASK1** (Q39255)	Cys-37, Cys-118 S-nitrosylation	Enhances interactions	(Iglesias et al., 2018)
*Arabidopsis*	**TIR1** (Q570C0)	Cys-40 S-nitrosylation	Enhances interactions	(Terrile et al., 2012)
*Arabidopsis*	**AHP1** (Q9ZNV9)	Cys-115 S-nitrosylation	Decreased activity	(Feng et al., 2013)
*Arabidopsis*	**SAMS** (P23686)	Cys-114 S-nitrosylation	Inactivation	(Lindermayr et al., 2006)
*Tomato*	**ACO1** (P05116)	Cys-60 S-sulfhydration	Decreased activity	(Jia et al., 2018)
*Tomato*	**ACO2** (P07920)	Cys-S-sulfhydration	Decreased activity	(Jia et al., 2018)
*Tomato*	**NOR TF** (Q56UP6)	Met-138 oxidation	Decreased the DNA binding activity	(Jiang et al., 2020)
*Arabidopsis*	**JAZ1** (Q9LMA8)	Cys-229 S-nitrosylation	Enhances interactions	(Pande et al., 2022)
*Arabidopsis*	**SABP3** (P27140)	Cys-280 S-nitrosylation	Lower affinity to SA binding and inhibition of carbonic anhydrase activity	(Wang et al., 2009)
*Arabidopsis*	**NPR1** (P93002)	Cys-156 S-nitrosylation	Enhances oligomeric state	(Tada et al., 2008)
*Arabidopsis*	**TGA1 TF** (Q39237)	Cys-260, Cys-266 S-nitrosylation	Cys overoxidation protection and increased the DNA binding activity	(Lindermayr et al., 2010)
*Arabidopsis*	**TGA1 TF** (Q39237)	Cys-260, Cys-266 S-glutathionylation	Cys overoxidation protection and increased the DNA binding activity	(Lindermayr et al., 2010)

## 7 Role of redox-based PTMs on photosynthetic metabolism, photorespiration and Calvin-Benson cycle

The overall photosynthesis process, including the biosynthetic pathways of elements from the photosynthetic apparatus, as well as complementary metabolic pathways, such as photorespiration and the Calvin-Benson cycle, have been described under redox regulation by multiple studies.

In *shorgum*, the photosynthetic enzyme C4 phosphoenolpyruvate carboxylase (PEPCase) is inactivated *in vivo*, under salinity stress, and *in vitro*, by carbonylation of multiple residues, due to oxidative stress induced by high concentrations of ROS. However, both *in vivo* and *in vitro*, PEPCase can be S-nitrosylated, a PTM that has no significant impact on its catalytic activity, although it does attribute to the enzyme resistance to carbonylation, preserving its activity under stress conditions. This finding suggests that PEPCase S-nitrosylation is a mechanism of salinity stress tolerance (Baena et al., 2017). In spinach, the carbonylation of its phosphoribulokinase (PRK) enzyme also induces the inactivation of its catalytic activity (Mano et al., 2009; Mano et al., 2014). Similarly, in *Arabidopsis*, its sedoheptulose-1,7-bisphosphatase (SBPase) and ribulose-1,5-bisphosphate carboxylase (RuBisCO) enzymes have been described as carbonylation targets, a PTM that induces their catalytic inactivation (Liu et al., 2012b; Lounifi et al., 2013). In *Brassica*, the RuBisCO enzyme also suffers redox regulation by means of Cys S-nitrosylation, causing an inhibition of its carboxylase activity (Abat and Deswal, 2009).

The oxidation of Trp to N-formylkynurenine by means of ROS has been described in two spinach proteins, linked to photosynthetic metabolism: the Trp352 residue of the CP43 subunit of PSII protein, and the Trp132 residue of the Lchb1 protein. These modifications have been associated to a mechanism of protein turnover to preserve the functionality of the photosynthetic apparatus under environmental stress conditions (Anderson et al., 2002; Rinalducci et al., 2005; Rinalducci et al., 2008). In *Arabidopsis*, Mg-protoporphyrin IX methyltransferase (CHLM), an enzyme linked to the biosynthesis of chlorophylls, is subject to redox regulation by means of the oxidation of its Cys111 and Cys115 residues. Both oxidations decrease this enzyme’s methyltransferase activity (Richter et al., 2016).

In *Arabidopsis*, the catalytic activity of the glyceraldehyde 3-phosphate dehydrogenase (GAPDH) enzyme increases by 60% due to the sulfhydration of its Cys residues (Aroca et al., 2015; Palma et al., 2020), and it is reversibly inhibited by S-nitrosylation of its catalytic Cys149, which is mediated by NO· donors (Zaffagnini et al., 2013). However, in cucumber, its homologous GAPDH enzyme decreases its catalytic activity when it undergoes Cys S-nitrosylation (Niu et al., 2019). Similar consequences cause Cys S-nitrosylation of the glycolate oxidase (GO) enzyme in peas (Ortega-Galisteo et al., 2012). In this same plant, other proteins have been described as susceptible to redox-based PTMs. Hydroxypyruvate reductase (HPR) can undergo nitration in its Tyr198 residue, with the consequent decrease in its catalytic activity (Corpas et al., 2013). Similarly, Tyr205 nitration of the sunflower carbonic anhydrase (β-CA) enzyme under high temperature abiotic stress conditions, also decreases its catalytic activity by 43% (Chaki et al., 2013).

The consequences that have been described in photosynthetic metabolism and enzymes linked to photorespiration and Calvin-Benson cycle, are related to the decrease/inhibition of their metabolic activity, or may even force the proteolysis of elements of the photosynthetic apparatus and their turnover, as a result of an excessive amount of redox-based PTMs on their residues. These discoveries demonstrate that photosynthetic metabolism and complementary pathways are subject to strict redox regulation, whose main consequences lead to a decrease in photosynthetic functionality, as well as a decrease in the catalytic activity of the enzymes that compose it. The exposure to abiotic stresses is known to disrupt the proper functioning of photosynthesis, but redox inactivation suggests that, under these conditions, cellular metabolism needs to be restructured, and energy sources need to be redirected to other metabolic pathways that allow the plant to survive.

The information described on the PTMs of photosynthetic metabolism, photorespiration and Calvin-Benson cycle-related proteins is shown in **Table 4**.

**Table 4:** PTMs and their effect on proteins involved in photosynthetic metabolism, photorespiration and Calvin-Benson cycle.

Plant	Protein (Uniprot accession number)	PTM	Effect	Reference
*Sorghum*	**PEPCase** (P15804)	Carbonylation	Inactivation	(Baena et al., 2017)
	**PEPCase** (P15804)	Cys-S-nitrosylation	Protection against carbonylation	(Baena et al., 2017)
*Spinach*	**PRK** (P09559)	Carbonylation	Inactivation	(Mano et al., 2009; Mano et al., 2014)
*Arabidopsis*	**SBPase** (P46283)	Carbonylation	Inactivation	(Liu et al., 2012b)
*Arabidopsis*	**RuBisCO** (O03042)	Carbonylation	Inactivation	(Lounifi et al., 2013)
*Brassica*	**RuBisCO** (P48686)	Cys-S-nitrosylation	Inhibition of carboxylase activity	(Abat and Deswal, 2009)
*Spinach*	**CP43** (P06003)	Trp-352 oxidation	Inactivation/Turnover	(Anderson et al., 2002)
*Spinach*	**Lchb1** (P12333)	Trp-132 oxidation	Inactivation/Turnover	(Rinalducci et al., 2005)
*Arabidopsis*	**CHLM** (Q9SW18)	Cys-111 oxidation	Decreased activity	(Richter et al., 2016)
	**CHLM** (Q9SW18)	Cys-115 oxidation	Decreased activity	(Richter et al., 2016)
*Arabidopsis*	**GAPDH** (P25858)	Cys-S-sulfhydration	Increased activity	(Aroca et al., 2015)
	**GAPDH** (P25858)	Cys-149 S-nitrosylation	Inactivation	(Palma et al., 2020)
*Cucumber*	**GAPDH** (A0A0A0K8C1)	Cys-S-nitrosylation	Decreased activity	(Niu et al., 2019)
*Pea*	**GO** (-)	Cys-S-nitrosylation	Decreased activity	(Ortega-Galisteo et al., 2012)
*Pea*	**HPR** (-)	Tyr-198 nitration	Decreased activity	(Corpas et al., 2013)
*Sunflower*	**β-CA** (A0A251ULA9)	Tyr-205 nitration	Decreased activity	(Chaki et al., 2013)

## 8 Conclusions and future perspectives

Different plant abiotic stresses, including salinity, extreme temperatures, drought, and heavy metal toxicity, among others, are the main adverse factors that limit plant development and decrease crop yields globally. For this reason, from all areas of study in the plant world, many approaches intended to decipher the physiological, biochemical and molecular mechanisms by which plants acclimatize to unfavorable conditions and tolerate abiotic stress exposure, or the simultaneity of different types of abiotic and biotic stresses.

The plants’ perception of abiotic stresses induces different molecular response mechanisms, among which we highlight the overproduction of reactive chemical species, mainly ROS, RNS and RSS. These molecules are present in all plant subcellular compartments, either by *in situ* production or through diffusion, and induce both damages to cellular components and biomolecules, and redox modulation/regulation of metabolism by means of PTMs in specific proteins. These redox-based PTMs mainly include Cys oxidation and S-nitrosylation, Tyr nitration, carbonylation, Met oxidation, Trp oxidation and Cys S-sulfhydration. PTMs can be produced in susceptible residues of specific proteins, exclusively or in combination with other modifications, within the same protein and, even, the same residue, multiplying the redox-based proteoforms of the plant proteome and increasing the complexity of cell signaling processes.

Recent research has elucidated how specific proteins, belonging to specific metabolic pathways, such as those involved in energy production, amino acid biosynthesis, cell signaling events or the global photosynthetic process, modify their catalytic activity by hosting some type of redox-based PTMs. However, most of this knowledge has been obtained by *in vitro* tests, following speculations obtained from other experimental results.

In addition, and as mentioned above, when two or more abiotic stresses act together, the plants’ response is very specific and cannot be deduced from their response against a single stress. In this sense, there is a very limited information in the literature on how redox-based PTMs affect plant metabolism under some type of simple abiotic stress, mainly salinity or high temperatures. However, there is absolutely no information on how these redox-based PTMs affect the plant’s response to abiotic stress combination. Therefore, it is necessary to increase the research and characterization of new target proteins that are susceptible to modulation by redox-based PTMs, and to elucidate their function in the response of plants to abiotic stress. In addition, new lines of research are needed to delve into the identification of PTMs that give rise to new proteoforms with new functionalities induced by the combination of abiotic stresses, which would open new fields of knowledge in the identification of the mechanisms of plant tolerance to abiotic stress, in order to increase the resilience of crops to climate change.

## Author contributions

JMM-G wrote the manuscript and designed the figures and tables. MP-H, SEM-L an LA contributed to the writing, editing and literature updating. RMR supervised, corrected and contributed with the writing and editing of the manuscript. All authors contributed to the article and approved the submitted version.

## Funding

This research was supported by the Ministry of Economy and Competitiveness from Spain (Grant No. PGC2018-09573-B-100) to RMR; by the Spanish National Research Council (CSIC) (JAEINT_21_01293) to SEM-L; by University of Murcia Ph.D. contracts (Registry number 109144/2022) to JMM-G; and by the Ministry of Science and Innovation of Spain (Grant No. FPU20/03051) to MP-H (Murcia, Spain).

## Acknowledgments

We sincerely acknowledge Mario G. Fon for proofreading the manuscript. We also apologize to all authors of papers not mentioned in this manuscript due to space limitations.

## Conflict of interest

The authors declare that the research was conducted in the absence of any commercial or financial relationships that could be construed as a potential conflict of interest.

## Publisher’s note

All claims expressed in this article are solely those of the authors and do not necessarily represent those of their affiliated organizations, or those of the publisher, the editors and the reviewers. Any product that may be evaluated in this article, or claim that may be made by its manufacturer, is not guaranteed or endorsed by the publisher.
